# The Use of a Theory of Change Model to Guide the Implementation of a Comprehensive Surgical Specialty Training Program in Equatorial Guinea

**DOI:** 10.5334/aogh.4477

**Published:** 2024-07-16

**Authors:** Robert Memba, Juan Carlos Puyana, Martha Grayling, Carme Climent, Patrícia Martínez, Eunice Blanco, Jordi Rigueiro, David Suárez, Guillem Viscasillas, Emma Fortea, Olga Roman, Daniel Gracia, Francesc Feliu, Silvano Nve, Rosa Jorba

**Affiliations:** 1 Más Que Salud (More than Health), Non-Governmental Organization, Spain; 2 Rovira i Virgili University (URV), Pere Virgili Institute for Health Research (IISPV).; 3 Global Health Surgery Department, University of Pittsburgh, US; 4 Institute of Global Surgery, Royal College of Surgeons in Ireland, Dublin; 5 Pediatrics Department, University General Hospital of Catalonia, Spain; 6 Orthopedics and Trauma Surgery Department, Parc Taulí University Hospital, Spain; 7 Social Work Department, Vall d’Hebron University Hospital, Spain; 8 INnGEAUDIO (Innovation and Engineering for Hearing), Spain; 9 Otorhinolaryngology Department, Althaia Manresa University Healthcare Network, Spain; 10 Dentistry Department, Dentalògic Dental Clinic, Spain; 11 Gynecology and Obstetrics Department, Manacor Hospital, Spain; 12 General Surgery Department, University Hospital of Tarragona Joan XXIII, Spain; 13 Anesthesiology Department, Bata General Hospital, Equatorial Guinea

**Keywords:** global surgery, surgical education, specialist training program, surgical trainees

## Abstract

*Background:* Equatorial Guinea (EG) is located on the African west coast, with only 0.4 trained physicians per 1,000 resident population. The country has one medical school and there is no specialist training program. From 2000 to 2022, 524 doctors have received their medical degree. However, the number of national surgical specialists in the entire country is currently 42.

*Objective: Formación Especializada Sanitaria en Guinea Ecuatorial* (FES Guinea) is a program specifically aimed at designing and implementing a long-term national surgical specialist training program.

*Methods: Más Que Salud* (+QS), which means “More than Health” in Spanish, is a nonprofit organization leading the FES Guinea program. We used the theory of change (ToC) framework to evaluate the work accomplished and implement subsequent phases. The initial phase (A) included a needs assessment and mapping of available resources. An intermediate phase (B) started with a memorandum of understanding to implement a Train the Trainer program. The consolidation phase (C) consists of educational interventions and future advanced training projects.

*Findings:* The ToC model allowed us an analyses of initial and intermediate phases. The needs assessments and resources mapping were executed while several scientific meetings and workshops were given. Scholarships to support specialist training abroad benefited six physicians in a diverse set of surgical disciplines. A regulatory commission to implement the FES Guinea program and the National Medical Council of EG were created. Working directly with the EG Ministry of Health, +QS codesigned a National Health Development Plan that began implementation in 2021 to continue until 2025.

*Conclusions:* The ToC model allowed us to predict the current and future potential effects of FES Guinea on surgical workforce development in EG. This is a unique surgical training program, which combined effective initiatives spearheaded initially by an NGO that successfully incorporated both local health and academic authorities, ensuring sustainability.

## Background and Objective

Equatorial Guinea is the only Spanish-speaking country in Africa. Following its independence from Spain in 1968, the nation’s health system underwent many changes. To date, overall access to healthcare in EG continues to be critically low, especially in rural areas [1]. According to the World Bank, EG has only 0.4 trained physicians per 1,000 resident population [2]. Worker shortages and a low number of qualified human resources personnel undermines efforts to improve EG’s current health system. Unfortunately, objective data regarding health outcomes such as disease prevalence, mortality, and healthcare workforce availability is limited. Data on the provision of surgical services is particularly scarce [1, 3, 4].

The only university in the country is the National University of Equatorial Guinea (UNGE). It was created and accredited by the Ministry of Education and Science of EG in January 1995. The School of Medical Sciences of the UNGE was founded five years later, in August 2000 [5]. The School of Medicine has an accreditation program to train physician “generalists” in the medical field. EG currently does not have any specialist training programs available. Consequently, there are no surgical specialty training programs, and a centralized accreditation process for currently practicing surgeons in EG is nonexistent. Continuing education opportunities for general medicine graduates are routinely not available, either [4].

From 2000 to 2022, 524 doctors have received their medical degree at the UNGE. During this period, 65 doctors (12.4%) have had access to a postgraduate specialty by training abroad (mainly in Cuba and occasionally in Senegal, Morocco, Ghana, Gabon, or South Africa). Due to the paucity of trained surgical specialists, the EG Ministry of Health and Social Welfare hires and provides salaries for expatriate surgeons to work and offer surgical services in EG. Unfortunately, the number of native surgical specialists is low due to limited access to these international opportunities. Currently, there are no standardized application processes or policies to allow a fair system based on performance on a qualifying examination or previous curriculum achievements. Although many graduates actively seek opportunities for training abroad, very few individuals are successful in obtaining such training.

Table 1 shows the current surgical workforce of EG. Interestingly, these data include anesthesiologists and dentistry specialists, as they are all members of the *Sociedad Científica Ecuatoguineana de Cirugía* (SOECIR), the Scientific Society of Equatoguinean Surgeons. Expatriate surgical specialists account for 67.5% of the 157 total surgical specialists for the entire country. Without counting dentists and anesthesiologists, there are only 42 national surgical specialists.

**Table 1:** Membership of SOECIR by surgical specialty and nationality.

SPECIALTY	EXPATRIATES	NATIVES	TOTAL
General Surgery	29	14	43
Obstetrics and Gynecology	25	14	39
Orthopedics and Trauma surgery	10	4	14
Urology	6	2	8
Neurosurgery	2	4	6
Anesthesiology	15	7	22
Dentistry and Dental Surgery	4	2	6
Otorhinolaryngology	3	1	4
Ophthalmology	6	2	8
Plastic Surgery	2	0	2
Oral and Maxillofacial Surgery	2	0	2
Angiology and Vascular Surgery	2	1	3
Total	106	51	157

Augmenting the surgical workforce in EG is a priority in the goal toward improving current gaps in healthcare coverage [6–8]. +QS is a nonprofit organization founded in 2007 with the main objective of improving health systems in EG [9]. +QS has embarked on a surgical specialty training program called the *Formación Especializada Sanitaria en Guinea Ecuatorial* (FES Guinea). From the very beginning of its conception, FES Guinea was designed and tailored to the local reality and the available educational resources in EG, by creating solid partnerships with UNGE and the EG Ministry of Health and Social Welfare. +QS was instrumental in establishing such partnerships and has been actively working in EG since 2007 with the ultimate objective of increasing the surgical workforce. +QS strategy is to offer to EG healthcare practitioners a variety of surgical training activities and educational interventions. FES Guinea is specifically aimed at designing and implementing a long-term national surgical specialization training program.

## Methods

**Figure 1:**
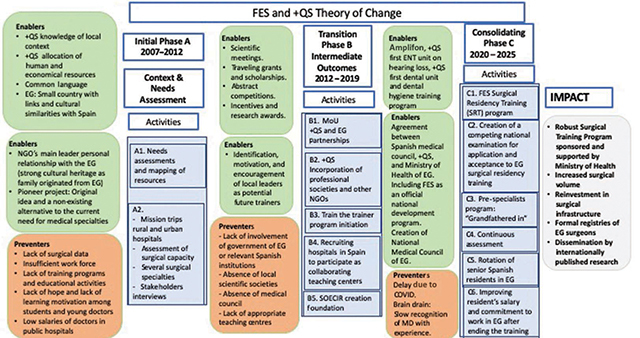
ToC model for FES.

We applied a theory of change framework [10] designed to evaluate the work accomplished by FES over the last 10 years and to guide FES Guinea’s intermediate and long-term objectives, as the combined efforts of +QS and key stakeholders in EG move into the future with new and more robust collaborative training and educational programs. The initial phase (A) included a needs assessment and familiarization phase (2007–2012); the subsequent intermediate phase (B) included several surgical training activities (2012–2019); and the more formal implementation and consolidation phase (C) currently being deployed, as well as future planning of a long-term training program (2020–present), are described and analyzed under the proposed ToC framework [11, 12]. Figure 1 shows the theory of change model projecting impacts of FES Guinea interventions.

## Theory of Change Phases

### Initial phase (A)

***Needs assessment and mapping of available resources:*** From 2007 to 2014, a number of preliminary visits and surgical missions were undertaken by +QS staff and collaborators in EG. Field visits and deployment of surgical teams were involved in providing care and engaging in a number of basic surgical activities in several regions of the country, including both rural district hospitals and the tertiary central university (Bata General Hospital). Surgeons representing +QS, in combination with local surgeons, provided surgical care in several disciplines, including general surgery, gynecology and obstetrics, orthopedics and trauma, dental surgery, otorhinolaryngology, ophthalmology, and plastic surgery. During these exploratory visits, assessment interviews were conducted with many stakeholders, including local surgeons, policymakers, members of the UNGE and the Ministry of Health, members of the community, patients, and medical students. Since 2009, +QS conducted interviews with both medical and surgical providers in EG, seeking to learn about the nuances of healthcare infrastructure in EG.

### Intermediate phase (B)

***Memorandum of understanding (MoU) to move forward:*** In February 2012, +QS and the UNGE signed the first formal agreement to work together. +QS committed to allocate human resources and organize educational activities targeting medical students and recent UNGE graduates (generalists) as well as physicians working as surgeons without formal surgical training. +QS was also successful in expanding the partnership with EG by bringing other Spanish professional associations to collaborate in these preliminary educational projects. The Dentistry School of the University of Barcelona, the International University of Catalonia (UIC), the General Spanish Medical Council (CGCOM), Amplifon (the world leader in hearing-care retail), and Quironsalud (Spain’s largest private hospital group) joined +QS and deployed many short-term and midterm academic activities in EG. From 2012 to 2019, seven scientific meetings organized by +QS in collaboration with UNGE took place in EG. These meetings included lectures, seminars, abstract competitions for oral presentations (for local scientific contributions), and simulation-based suture-surgical skills workshops.

***The Train the Trainer program:*** was also instituted during this intermediate phase and involved providing grant support for EG surgeons to visit hospitals in Spain for a two-month period in order to learn how surgery training programs work and to improve their surgical skills and knowledge. During these rotations, the selected surgeons were fully engaged in the activities of an academic hospital, participating in ward rounds, multidisciplinary meetings, workshops, and other teaching activities [13], including scrubbing and assisting in a variety of surgical procedures.

### Consolidation phase and future projects / interventions (C)

***FES Guinea:*** A plan to institute a five-year surgical residency training program in EG has been developed. This plan was created with mutual cooperation and participation of members of SOECIR. SOECIR is the only medical scientific society in EG, and it is composed of members from several disciplines and specialties, including anesthesiology, angiology and vascular surgery, dentistry and dental surgery, general surgery, neurosurgery, obstetrics and gynecology, ophthalmology, otorhinolaryngology, orthopedics and trauma surgery, and urology.

***Brief description of the Surgical Residency Training (SRT) program components****:* The basic surgical principles will be taught in structured programs under the tutorship of both EG and Spanish consultants. These concepts will be taught by surgeons during their periods in Spain and under Equatoguinean surgeons during their local rotations. A combination of bedside teaching, clinical sessions, surgical videos, slide projections, surgical sessions, workshops, audits, and personal tutor meetings will be delivered to impart knowledge and technical skills to trainees. Surgical residents will be expected to attend update courses, revision programs, and a number of prescribed skills courses, as well as research training activities. The UIC is committed to the project and has offered a dedicated facility for simulation; therefore, a surgical skills course with training in suturing and anastomosis techniques will also be included in the program. Table 2 shows an example of a preliminary five-year training rotation schedule for a surgical resident. The final rotation will always take place in EG and the official specialist certificate will be granted by the EG government.

**Table 2:** Example of General Surgery rotation schedule.

ACADEMIC YEAR	JULY–DECEMBER (SPAIN)	JANUARY–JUNE (EQUATORIAL GUINEA)
**2025-2026**	**POSITION ASSIGNMENT** University General Hospital of Catalonia Abdominal Wall Surgery 3 months Colorectal Surgery 3 months	Bata General Hospital General Surgery 3 months *La Paz* Bata General Emergencies 3 months
**2026-2027**	Mútua Terrassa University Hospital Neurosurgery 1 month Urology 1 month Otorhinolaryngology 1 month *Parc Taulí* University Hospital. Orthopedics and Trauma Surgery 1 month Angiology and Vascular Surgery 1 month *Dentalògic* Dental Clinic Dental Surgery 1 month	Bata General Hospital Obstetrics and Gynecology 1 month *La Paz* Bata Ophthalmology 1 month Anesthesiology 1 month Guineasalud General Surgery 2 months Guineasalud Intensive Care Unit 1 month
**2027-2028**	University Hospital Tarragona Joan XXIII Hepato-Pancreato-Biliary Surgery 2 months Endocrine, Abdominal Wall, and Gastric Surgery 2 months Colorectal Surgery 2 months	Loeri Comba Hospital General Surgery 3 months Malabo General Hospital General Surgery 3 months
**2028-2029**	Vall d’Hebron University Hospital. Hepato-Pancreato-Biliary Surgery 3 months Endocrine and Esophagogastric Surgery 3 months	*La Paz* Malabo General Surgery 3 months Malabo General Hospital General Surgery 3 months
**2029-2030**	Vall d’Hebron University Hospital Colorectal Surgery 3 months Free rotation 3 months	Free rotation 3 months *Virgen de la Guadalupe*. General Surgery **GENERAL SURGERY CERTIFICATE DELIVERY**

***Creation of a competing national examination for application and acceptance to EG surgical residency training:*** +QS was successful in cocreating a regulatory commission with participation of members of the Ministry of Health and Social Welfare of EG and the Ministry of Education of EG. This partnership is fully committed to establish a national evaluation system that will be required, as access to a surgical resident position will be highly competitive. This commission will have members of +QS as well as several representatives of the local authorities. This evaluation will include a competency exam, a review of overall merit and accumulated experience from the curriculum vitae, and a formal panel interview. The trainee positions will be granted by the Ministry of Health and Social Welfare of EG, while the final surgical training accreditations will be awarded by both the Ministry of Education of EG and the Ministry of Health and Social Welfare of EG. The trainees (residents) will be employed by the Ministry of Health and Social Welfare of EG. The EG government is fully committed to supply their salaries throughout the full length of their training period. The only requirement to apply will be that applicants must be graduates of the medical school of UNGE.

***Accreditation of hospitals as teaching centers in EG:*** Several hospitals, both from the public and the private sectors, will be accredited by the same regulatory commission to become teaching centers when meeting the preestablished standard criteria.

***Co-participation of teaching hospitals in Spain to offer extramural rotations to EG trainees:*** Accreditation criteria will also be applied to hospitals in EG and in Spain. The trainees will rotate in alternating periods of six months between EG and Spain to avoid higher rates of emigration of Equatoguinean trainees who will be highly trained surgeons.

***Pre-specialists program—“grandfathered in****”:* There are a number of doctors who have been working for many years in EG, providing specialized surgical care even though they did not complete formal training in those areas. Some of these self-trained specialists have been working for more than 10 years in their own disciplines. These senior and experienced clinicians will be grandfathered in for the purposes of our training program. This group of clinicians will be offered the opportunity to spend a rotation in a Spanish hospital, and they will write a certification examination as part of the prerequisite for grandfathering in. These surgeons’ knowledge and clinical trajectories will then be recognized, as they represent an important asset to the EG academic institutions and hospitals where they practice. They will continue to provide support and teaching to the new graduates of our combined training program.

***Continuous assessment of surgical trainees:*** Progress of training will be monitored through the compulsory use of an approved logbook, periodic meetings with the designated tutors whose duty will be to assure and evaluate adherence to the curriculum, and annual (in-service) exams to assess acquired knowledge. Simulation scenarios will be created and included in this program for continuous assessment and evaluation of training. Context-appropriate scenarios will be created to ensure that trainees are exposed to unusual diseases’ characteristics of the EG geographical region. These simulation scenarios are aimed to ensure that candidates spending time abroad while being trained under the collaboration with Spanish hospitals training sites will still be able to identify and manage conditions typical of the burden of disease in EG. By the same token, trainees will also be exposed to high-income settings and will have the opportunity to participate in advanced surgical experiences that may not be otherwise accessible in many areas of EG. By doing so, they will have the opportunity to familiarize themselves with standards of care and logistical support (e.g., pathology, blood banking, Intensive Care Unit care) scenarios as part of the overall surgical education.

In addition, the regulatory commission will periodically evaluate each of the teaching centers to validate their academic capacity.

The theory of change model [12] was applied retrospectively to analyze FES Guinea’s initial and intermediate phases. It will also be used to guide—in a prospective evaluative program—the immediate, intermediate, and long-term effects of the upcoming subsequent activities and new interventions, which will be driven by +QS and EG local partners. FES Guinea objectives and innovative interventions aim at (1) continuing to enhance educational activities, (2) creating and implementing a nationwide surgical curriculum, and (3) formalizing a certification process to provide a solid and eventually official infrastructure for surgical training in Equatorial Guinea. The ToC model will allow us to predict and project the current and future potential effects of FES on surgical workforce development in Equatorial Guinea (Figure 1).

## Results

***Results and outputs—initial phase:*** From 2012 to 2019, seven scientific meetings organized by +QS in collaboration with UNGE took place in EG. These meetings included lectures, seminars, and simulation-based suture workshops. In 2012 as part of these scientific meetings, +QS coordinated the first live, real-time televised transmission of a surgical intervention performed in a hospital in EG as a means of illustrating surgical techniques to a large audience. More than 400 participants were registered and participated in the different scientific meetings. The participants were qualified specialists and junior general doctors from all over EG, along with medical students. The lectures were led by both Spanish and Equatoguinean doctors.

The team of visiting surgeons performed basic surgical procedures (cholecystectomies, hernia repairs, and cataract surgeries) and all the participants were able to see the operations on a big screen and talk to the surgeon. These live interactions with the surgeons while they were doing the cases was unprecedented in EG. This learning experience was very successful: it was positively reviewed by the trainees and aided tremendously to demonstrate +QS’s serious commitment to teaching. Soon after this trip, UNGE asked +QS to sign the first official collaborative training agreement.

During the COVID-19 pandemic, an online conference, including webinars and surgical videos, was offered as a continuing educational resource. How to focus on basic surgical care and surgical emergencies in such a complicated period was emphasized during that conference [14]. In February 2023, our first visit after the pandemic, significant progress was made to begin final planning for implementing a residency program in pediatrics, followed by general surgery.

Equatoguinean doctors were invited to submit material for oral presentations and posters during the conferences. Abstract submission was encouraged and all material was accepted. On average 20–25 abstracts were presented per annual conference. They included case reports, short series, bibliographic reviews, and a few retrospective studies. Some were accepted for oral presentations, others for posters. The topics included specific conditions in EG such as hernia complications, trauma injuries, and abdominal infections. The best scientific oral presentations were rewarded with grants. These awards consisted of financial support for traveling to Spain and spending time in clinical rotations in several hospitals. To date, six doctors have benefited from these surgical grants: two doctors visited general surgery departments; another two, otorhinolaryngology departments; and the other two doctors visited anesthesiology departments. All six doctors stayed in academic centers, having been involved in clinical practices and having received other additional teaching tools, and they all returned to EG.

***Results and outputs—intermediate phase:*** During the intermediate phase, +QS formed partnerships to promote dentistry services, hearing aid access, and basic pediatric care.

In 2016, in collaboration with Amplifon, +QS created the first otorhinolaryngology unit focused on hearing loss in EG. Two generalist practitioners currently work in this unit after having received basic training during two months in Spain. These doctors continue to receive online support from several Spanish otorhinolaryngology specialists. Those same specialists have continued to provide additional onsite supervision when they visit EG, which they have done once or twice per year since 2016.

A dental unit was also created by +QS in 2017. A two-year grant to train in dental hygiene was also given by +QS. A dental hygienist is working in the unit and training two other people to work as dental hygienists. There has not yet been an opportunity to train a dentist in this unit.

In addition, +QS supported one nurse when she applied for an official one-year postgraduate course in surgical nursing. After completing the course, this nurse went back to EG, where she is currently working in several hospitals as the only certified surgical nurse in the country. In total, four operating-room nurses from EG were awarded grants to train in Spanish hospitals by +QS.

***The creation of SOECIR:*** In 2019, practicing surgeons in Equatorial Guinea created the *Sociedad Científica Ecuatoguineana de Cirugía* (SOECIR), a society of surgeons dedicated to communication, collaboration, and the promotion of surgical training. SOECIR includes native and some expatriate surgeons. This society allows the collection of surgical workforce data that was previously less accessible. A member registry of practicing surgeons in EG, along with their specialties, their hospitals, and their nationalities, was obtained from the SOECIR database.

Currently, there are 51 native Equatoguinean surgeons who are practicing members of SOECIR. Over half (54.9%) of these surgeons specialize in general surgery or obstetrics and gynecology. There are 157 surgeons and 15 anesthesiologists in the SOECIR database, and 67.5% of members are expatriates. Expatriates outnumber native surgeons in every specialty besides neurosurgery. Table 1 shows the SOECIR members included in the database.

***Expected results and outputs—consolidation phase:*** The consolidation phase (C) has been progressively implemented since 2020. A commitment to mutual collaboration of key stakeholders, policymakers, and decision-makers has been readily achieved. Two events have been especially decisive in providing a robust infrastructure for the successful and sustainable implementation of the consolidation phase. First, the Ministry of Health and Social Welfare of EG assessed and accepted the proposal submitted by +QS to begin the specialist training programs with pediatrics, followed by general surgery, and included the program in the National Health Development Plan for 2021–2025 [15]. Second, an agreement between the Ministry of Health and Social Welfare of EG, +QS, and the Spanish CGCOM guaranteed that the trainees will have official access to the Spanish public and private hospitals network.

In 2021, +QS, the Ministry of Health and Social Welfare of EG, and CGCOM signed an official agreement toward the creation of the EG NMC (Equatorial Guinea National Medical Council). In February 2023, after an official meeting with the ministry of Health and Social Welfare of EG, the UNGE, and the CGCOM, the regulatory commission was created in order to implement the program in 2024. Finally, the EG NMC was created as a democratic nongovernmental association in March 2024. This official medical council will aim to regulate doctors by ensuring professional competence and continuous education, promoting appropriate conduct and ethics, and acting as a mediator in patient–doctor conflicts. The NMC will also manage training requirements for accreditation and will eventually facilitate healthcare data collection [16]. The technical office of the Ministry of Health is currently organizing an official meeting where +QS and the Ministry of Health will present the program to the president of the country or the prime minister in order to get the presidential approval necessary to implement the program and to guarantee a long-term investment, including a commitment to reallocate financial support to ultimately secure sustainability. The FES Guinea program has become a priority for the Ministry of Health and a road map has been described in detail, including a timetable to begin the functions of the regulatory commission and a date for the first round of examinations for June 2025.

## Discussion

Equatorial Guinea is a small, Spanish-speaking country on the west coast of Africa with severely limited access to surgical services [4]. Currently, a significant portion of the surgical care available in EG is provided by expatriate physicians trained outside EG. Native surgeons have been trained abroad by international trainers and foreign educators to perform surgery, without a formal nationwide residency program. Currently, there is not a standardized surgical training curriculum in EG, and there is not an organized surgical residency training program in any of the nation’s central hospitals. There are not enough trained surgeons in the country. Therefore, particularly in rural areas, some general practitioners are performing surgical procedures without any formal surgical training.

FES Guinea is a special project of the NGO +QS, aimed at providing a surgical specialty training program. The theory of change model [11] described here allows us to integrate the work done so far by +QS. It also provides a framework to project into the future the potential impact of planned interventions and the expansion of collaborative efforts, in order to anticipate intermediate and long-term outcomes.

The theory of change model [10] illustrates the multiple connections between surgical specialty training and improved access to surgical services, trainee accreditation, and tracking of health data.

Implementation of a sustainable health system and surgical system development in EG will benefit from dynamic and productive interaction between physicians and government officials. +QS has been successful in advocating to decision-makers and in gaining government support. Further advocacy work will facilitate the dissemination of accreditation systems for specialists and will enhance healthcare providers’ participation in future infrastructure development.

This pioneering NGO-led training program for surgeons in EG is unique and effective. Nonprofit organizations sometimes undermine local health systems by neglecting to strengthen local organizations, with the result of duplicating actions or planning unrealistic projects. In our experience, local stakeholders needed to be completely involved from the beginning of any international aid or collaborative educational training project [17]. To date, FES Guinea has been successful because of +QS’s deep knowledge of the local context, long-lasting field experience in EG, close collaboration with key stakeholders in EG, and having obtained the support and commitment of relevant institutions both in EG and in Spain.

The regulatory commission was cocreated among +QS leadership and the EG stakeholders and MoH representatives. It is planned for +QS to phase out its role and participation in this commission as the commission becomes autonomous and self-sustained. We envisioned that the role of the +QS will decrease gradually over the years. The involvement of +QS will be progressively reduced after every cyclical assessment of the results of the program.

During the periods of time when Equatoguinean trainees will be rotating in Spanish hospitals, senior Spanish trainees will rotate in hospitals of EG as part of their involvement with global surgery. The program design will result in a mutually beneficial exchange of residents between EG and Spain. Inclusion of global surgery in all high-income countries (HIC) surgical training programs was also recommended by the Lancet Commission’s follow-up report [7]. This recommendation was based on three statements: (1) High levels of interest in pursuing global surgery are common among surgical residents; (2) training is incomplete for any HIC surgical resident without the knowledge of the global context of surgical care and burden of disease; and (3) senior surgical residents have the capability of performing basic procedures under supervision, of engaging in local research projects, and of sharing youthful enthusiasm, all of which have proved to be helpful in low-and middle-income countries (LMICs) [18–23].

Currently, the government of EG is subsidizing salaries by paying expatriate specialists. These salaries are much higher than the standard compensation made to physicians. Many of the expatriates work exclusively in the private sector/hospitals, which was implemented relatively recently. Implementation of the long-term educational plan proposed by FES Guinea will represent major economical savings. Training local human resources personnel will be more cost-effective. Increasing the number of properly trained EG *native* surgeons will gradually reduce overall healthcare expenses without reducing the quality of surgical care provided [24].

***Limitations:*** The principal limitation of this project is the fact that the impact on the health system most likely will be seen in the medium term or long term. Therefore, it is critical to understand that some stakeholders may give up on the process. The second limitation is based on acknowledging that the program needs a long-term commitment of political and academic institutions. However, it is well known that these institutions’ leadership could change in the future. Accordingly, formal written agreements have been signed to increase the chances of continuity and success. A third limitation pertains to a current low capacity to prospectively collect data with accurate metrics to assess the impact of the training programs. The creation of robust health informatics platforms and data registries to track and evaluate the quality and effectiveness of the training programs, to provide material to maintain continuing educational activities, and, most importantly, to measure the impact of these trainees on patient outcomes and overall quality of care will be fundamental to demonstrate achievement of long-term objectives. There is no existing infrastructure for clinical-outcomes tracking in EG, as it stands. Among patients that have a known medical history, interactions with the healthcare system are recorded in paper notebooks that they bring with them to the hospital. Developing a centralized medical record-keeping system, starting with a surgical registry, is key for infrastructure development. Collecting data allows trainees and physicians to analyze and evaluate the results of educational interventions and will aid in advocating to decision-makers and policymakers for their patients. For the Ministry of Health of EG, outcomes data can provide a basis for future expansion of the infrastructure investment and international aid provision.

The MoH understands that FES Guinea might be one of the keys to address the main issues of the health system and therefore have put their trust in +QS to collaborate in the initial implementation. The commitment of both MoH and UNGE is to gradually assume total control of the project.

+QS work has been long and arduous; however, several medical awards have been granted to +QS as a result of the project design and its results to this date: the Joaquim Bonal for Solidarity Projects Award of the Medical Sciences Academy of Catalonia and the Balearic Islands in 2011, the Solidarity Award of the GAES company in 2016, the Global Health Award from the Quironsalud Foundation in 2020, and the Ubuntu Award from the Euro African International Forum in 2022. The funds obtained from those awards have been used to pay for the expenses of scientific meetings and doctor rotations in Spain.

## Conclusions

Prior to the FES Guinea project, there was no accreditation system or specialty training program for Equatoguinean surgeons. +QS partnered with the EG Ministry of Health and Social Welfare and with the UNGE to plan a surgical training program and annual research conferences, and with the EG National Medical Council to increase surgical capacity and infrastructure. The theory of change analysis for this intervention may serve as a reference point to anticipate outcomes as FES Guinea enters its implementation phase. Data collection will be critical in establishing patterns of surgical care and tracking patient outcomes.

We anticipate that long-term outcomes of the surgical specialty training program will include increased surgical volume, investment in surgical infrastructure, formal registries of EG surgeons, and dissemination through internationally published research.
